# International consensuses and guidelines on diagnosing and managing fungal endophthalmitis by the Asia-Pacific Vitreo-retina Society (APVRS), the Academy of the Asia-Pacific Professors of Ophthalmology (AAPPO), and the Asia-Pacific Society of Ocular Inflammation and Infection (APSOII)

**DOI:** 10.1186/s40662-025-00456-y

**Published:** 2025-10-13

**Authors:** Taraprasad Das, Nishant V. Radke, Ahmed B. Sallam, Andrew Chang, Andrzej Grzybowski, Bahram Bodaghi, Chi-Chun Lai, Harry Flynn Jr, Han Joo Cho, Hiroto Ishikawa, Hua Yan, Joveeta Joseph, Kuan-Jen Chen, Landon J. Rohowetz, Li Jia Chen, Liuxueying Zhong, Matthew P. Simunovic, Paisan Ruamviboonsuk, Prashanth Iyer, Robert F. Lam, Rupesh Agrawal, Vivek P. Dave, Xiangyu Shi, Dennis S. C. Lam

**Affiliations:** 1 Department of Vitreoretinal Services, Shantilal Shanghvi Eye Institute, Mumbai, India; 2 Anant Bajaj Retina Institute, Srimati Kanuri Santhamma Center for Vitreoretinal Diseases, LV Prasad Eye Institute, Hyderabad, Telangana India; 3 The Primasia International Eye Research Institute (PIERI), The Chinese University of Hong Kong (Shenzhen), Longxiang Boulevard, Longgang District, Shenzhen, 2001 China; 4 Department of Ophthalmology, Jones Eye Institute, University of Arkansas for Medical Sciences, Little Rock, AR USA; 5 Sydney Retina Clinic, Sydney Eye Hospital, University of Sydney, Sydney, NSW Australia; 6 Institute for Research in Ophthalmology, Foundation for Ophthalmology Development, Poznan, Poland; 7 Department of Ophthalmology, Pitié-Salpêtrière University Hospital, Sorbonne University, Paris, France; 8 Department of Ophthalmology, Linkou Main Branch, Chang Gung Memorial Hospital, Taoyuan, Taiwan, China; 9 Department of Ophthalmology, Chang Gung Memorial Hospital, Keelung, Taiwan, China; 10 Department of Ophthalmology, Bascom Palmer Eye Institute, University of Miami, Miami, FL USA; 11 Department of Ophthalmology, Kim’s Eye Hospital, Youngdeungpo-Gu, Seoul, South Korea; 12 J-CREST (Japan Clinical REtina STudy Group), Kagoshima, Japan; 13 Department of Ophthalmology, Hyogo Medical University, Nishinomiya, Japan; 14 Department of Ophthalmology, Tianjin Medical University General Hospital, Tianjin, China; 15 Laboratory of Molecular Ophthalmology and Tianjin Key Laboratory of Ocular Trauma, Tianjin Medical University, Tianjin, China; 16 School of Medicine, Nankai University, Tianjin, China; 17 Jhaveri Microbiology Centre, Brien Holden Eye Research Center, LV Prasad Eye Institute, Hyderabad, Telangana India; 18 College of Medicine, Chang Gung University, Taoyuan, Taiwan, China; 19 Department of Ophthalmology and Visual Sciences, The Chinese University of Hong Kong, Hong Kong, China; 20 Department of Ophthalmology and Visual Sciences, The Prince of Wales Hospital, Hong Kong, China; 21 Hong Kong Eye Hospital, Hong Kong, China; 22 Department of Ophthalmology and Visual Sciences, Lam Kin Chung. Jet King-Shing Ho Glaucoma Treatment and Research Center, The Chinese University of Hong Kong, Hong Kong, China; 23 Hong Kong Hub of Pediatric Excellence, The Chinese University of Hong Kong, Hong Kong, China; 24 State Key Laboratory of Ophthalmology, Guangdong Provincial Key Laboratory of Ophthalmology Visual Science, Zhongshan Ophthalmic Center, Sun Yat-Sen University, Guangzhou, China; 25 Save Sight Institute, Sydney Eye Hospital Campus, Sydney, NSW Australia; 26 Sydney Eye Hospital, Sydney, NSW Australia; 27 Retina Division, Department of Ophthalmology, Faculty of Medicine, Rajvithi Hospital, Rangsit University, Bangkok, Thailand; 28 Department of Ophthalmology, Caritas Medical Center, Hong Kong, China; 29 National Healthcare Group Eye Institute, Tan Tock Seng Hospital, Singapore, Singapore; 30 Yong Loo Lin School of Medicine, National University of Singapore, Singapore, Singapore; 31 Lee Kong Chian School of Medicine, Nanyang Technological University, Singapore, Singapore; 32 Singapore Eye Research Institute, Singapore, Singapore; 33 Duke-NUS Medical School, Singapore, Singapore; 34 Beijing Tongren Eye Center, Beijing Tongren Hospital, Beijing Ophthalmology and Visual Science Key Lab, Beijing Institute of Ophthalmology, Capital Medical University, Beijing, China; 35 The C-MER Dennis Lam & Partners Eye Center, C- International Care Group, Hong Kong, China; 36 Department of Ophthalmology, Chang Gung Memorial Hospital, Xiamen, China; 37 The C-MER (Shenzhen) Dennis Lam Eye Hospital, Shenzhen, China; 38 Eye Department, C+ Health CKJ (Shenzhen) Hospital, Luohu, Shenzhen, China

**Keywords:** Controversy, Consensus, AAPPO, APVRS, APSOII, Fungal endophthalmitis, Diagnosis, Management

## Abstract

Fungal endophthalmitis represents one of the most challenging intraocular infections to diagnose and manage in ophthalmology. Despite advances in diagnostic techniques and treatment options, numerous controversies persist regarding optimal approaches to this sight-threatening condition. Due to the low incidence and significant variation in the severity and time of presentations, no large-scale randomized controlled trials have been done. Therefore, identifying controversies and deliberating the best approach to diagnosing and treating fungal endophthalmitis by international experts would help establish consensus statements that can guide clinical practice. The Asia-Pacific Academy of Professors in Ophthalmology (AAPPO), Asia-Pacific Vitreo-Retina Society (APVRS), and Asia-Pacific Society of Ocular Inflammation and Infection (APSOII) saw this critical gap and formed an international panel of experts comprising 24 experts to establish 20 consensus statements. While there is consensus on the need for early diagnosis and prompt administration of antifungal therapy, there are conflicting views on the optimal diagnostic approach to be taken, the role and timing of performing vitrectomy, and the use of systemic antifungal agents. A particularly contested topic is the role of corticosteroids. In establishing the 20 consensus statements, these thus serve as guidelines for diagnosing and managing fungal endophthalmitis.

## Background

Globally, over 300 million people are affected by a severe fungal infection, and 25 million are at high risk of dying or losing their sight [1]. Two important ocular fungal infections are keratitis and endophthalmitis. The incidence of fungal endophthalmitis is higher in South Asia and Southeast Asia than in Europe and North America [2]. This has been noted to increase with the widespread use of immunosuppressive therapies and invasive procedures. Without a global incidence report and a randomized clinical trial, there is no universally accepted diagnosis and management protocol for fungal endophthalmitis. Therefore, we took the opportunity to identify areas of controversy and establish consensus on diagnosing and managing fungal endophthalmitis. The Asia-Pacific Vitreo-Retina Society (APVRS), the Academy of the Asia-Pacific Professors of Ophthalmology (AAPPO), and the Asia-Pacific Society of Ocular Inflammation and Infection (APSOII) have chosen “fungal endophthalmitis” as one of the targeted topics for consensus and controversy for 2025 because of the reasons mentioned above. Two of this manuscript's senior authors (TD and DSCL) were appointed to coordinate this consensus project. This manuscript synthesizes evidence-based, real-world practice recommendations from leading global experts to guide diagnosing and managing fungal endophthalmitis.

## Methodology

Further to appointing the two coordinators, the AAPPO, APVRS, and APSOII formed an international expert panel comprising 24 panelists from 12 countries/territories. A core group of three members (TD, DSCL, and NVR) was established to perform literature search and review on fungal endophthalmitis as well as prepare the first draft of the identified controversies and consensus developed with explanation and elaboration. These statements were organized into five categories: disease entity, clinical diagnosis, pathobiology including microbiology, treatment, and future development. Each panel member independently and anonymously reviewed each statement and provided comments to the core group. The core group then reviewed, evaluated the feedback and comments, revised, and sent out the second draft for further opinions. This process was repeated until the statements were finalized. Subsequently, each panel member voted on each statement anonymously using a five-point Likert scale, ranging from ‘strongly agree,’ ‘agree,’ ‘neutral,’ ‘disagree,’ to ‘strongly disagree.’ A consensus was reached when at least 75% of the experts voted either 'agree' or 'strongly agree' for a statement.

## Controversies and consensus statements

### Section 1. Disease entity

Fungal endophthalmitis accounts for approximately 15%–19% of all endophthalmitis globally. The incidence after an intraocular procedure (chiefly cataract surgery) is lesser in North America and Europe [3, 4] than in Asia [5, 6]. Fungal endophthalmitis is classified into exogenous and endogenous. The incidence of these two forms varies based on multiple patient factors, including underlying health conditions and risk factors such as recent surgeries or intravenous drug use. The reported incidence of postoperative, traumatic, and endogenous fungal endophthalmitis in India is 16.7%, 14.4%, and 18% of all endophthalmitis, respectively [7–9]. Fungal endophthalmitis, unless after trauma, usually presents as delayed onset endophthalmitis [10, 11]. The time to the onset of symptoms is an important differentiating feature. Acute bacterial endophthalmitis presents within days, and fungal endophthalmitis presents in weeks to months. It is usually indolent and may persist for a variable time. This contrasts with acute bacterial endophthalmitis, which presents as a single episode of severe inflammation [12, 13]. In addition to reduced vision, there would be a history of intraocular surgery or trauma, invariably with vegetative material (exogenous infection) or illness that also required intravenous fluid injection (endogenous infection). A history of long-time use of topical corticosteroids and/or broad-spectrum antibiotics to reduce recurrent and persistent redness and inflammation/infection is not uncommon. The inflammation typically worsens when steroids are tapered or discontinued. Outbreaks of fungal endophthalmitis after cataract surgery were described previously [14]. Traumatic fungal endophthalmitis often occurs after injury with vegetable matter, stone, or mud particles [13, 15]. The symptoms and signs develop earlier than post-procedure and endogenous endophthalmitis.

Patients with endogenous fungal endophthalmitis often present with underlying systemic disease (common, diabetes mellitus), chronic infection (common, respiratory, and urinary tract infection), recent hospitalization (many times with an indwelling catheter), recent surgery (common, gastrointestinal, and colon), and immunosuppression (common, malignancy, immunodeficiency syndrome). A break in the blood-retinal barrier and a disrupted Bruch’s membrane result in the spread of endogenous sources of fungal infection from chorio-capillaries into the vitreous. This results in acute suppurative inflammation of the vitreous. As a result, vitritis is the most consistent presentation in endogenous endophthalmitis, sometimes with characteristic lesions in the vitreous and/or retina. The most common sites of systemic infection are the liver (pyogenic liver abscess), lung (pneumonia), endocardium (infective endocarditis), and presumed cutaneous or pulmonary sources in intravenous drug users [1]. Intravenous drug use increases the risk of endogenous fungal endophthalmitis. In the USA, Europe, and Australia, intravenous drug use-related endogenous fungal endophthalmitis is typically caused by *Candida albicans* [16–20].

Acute fungal endophthalmitis after cataract surgery is rare, but is a possibility. In an Indian study, nearly half to three-quarters of cases of fungal endophthalmitis were acute (time to onset of symptoms 30 days or less), and it was distributed nearly equally among three common fungi, *Aspergillus*, *Candida,* and *Fusarium* spp. [21].

The incidence of fungal endophthalmitis from large studies that analyzed at least 100 patients is listed in Table 1 [21–28]. *Aspergillus* spp. and *Candida* spp. are the predominant causative fungi in exogenous and endogenous fungal endophthalmitis.

**Table 1:** The incidence of fungal endophthalmitis has been reported in larger studies worldwide

	Authors	Period	Country	No. of eyes	Predominant fungal pathogen	Percentage of fungal endophthalmitis
Exogenous						
	Das et al. [21]	2005–2020	India	3830 Culture-proven	*Aspergillus* spp. 39.0% *Fusarium* spp. 15.9% *Candida* spp. 15.1%	19.1% Postoperative: 46.9% Traumatic: 35.6%% Endogenous: 17.5
	Yang et al. [22]	2013–2020	China	383 Culture-proven	Post-traumatic With IOFB: * Aspergillus* spp. 2.6% * Fusarium* spp. 2.6% Without IOFB: * Aspergillus* spp. 5.5% * Fusarium* spp. 2.7% * Candida* spp. 1.6% * Mucor* spp. 1.6%	With IOFB: 7.9% Without IOFB: 15.6%
	Chakrabarti [23]	1992–2005	India	113 Culture-proven	*Aspergillus* spp. 54.4% *Candida* 24.6% *Melanized* fungi 10.6%	Postoperative: 46.9% Post-trauma: 42.5% Endogenous: 10.6%
Endogenous						
	Schimel et al. [24]	2000–2011	USA	448 Culture-proven	*Candida albicans* 5.8%	15.8%
	Chen et al. [25]	2005–2024	China	177	N/A	12.4%
	Cho et al. [26]	2006–2013	USA, South Korea	174	*Candida* spp. 22.7% *Aspergillus* spp. 3.1%	All: 25.8% USA: 34.3% Korea: 16. 4%
	Dave et al. [27]	2006–2018	India	173 Culture-proven	*Candida* spp. 4.6% *Aspergillus* spp. 4.0% *Fusarium* spp. 1.7%	13.9%
	Kim et al. [28]	2006–2018	South Korea	152 Candidemia	*Candida* spp. 19.1%	19.1%

*IOFB* = intraocular foreign body; *N/A* = not available

**Consensus Statement 1.1:** *Fungal infections of the eye occur less frequently than bacterial infections. It is usually a delayed presentation. [Consensus score: 100% (strongly agree: 80%; agree: 20%; neutral: 0%; disagree: 0%; strongly disagree 0%)].*

**Consensus Statement 1.2:** *Endogenous fungal endophthalmitis is as common as exogenous endophthalmitis. The common systemic diseases are the liver, the lungs (pneumonia), and the endocardium. [Consensus score: 50% (strongly agree: 15%; agree: 35%; neutral: 15%; disagree: 35%; strongly disagree 0%)].*

**Consensus Statement 1.3:** *Acute fungal endophthalmitis after an intraocular procedure is also a possibility. [Consensus score: 95% (strongly agree: 40%; agree: 55%; neutral: 5%; disagree: 0%; strongly disagree 0%)].*

**Consensus Statement 1.4:** *Traumatic fungal endophthalmitis usually results from vegetative matter or soil injuries and presents earlier than other forms of fungal endophthalmitis. [Consensus score: 100% (strongly agree: 70%; agree: 30%; neutral: 0%; disagree: 0%; strongly disagree 0%)].*

**Consensus Statement 1.5:** *Vitritis is the most consistent clinical presentation in endogenous fungal endophthalmitis and may be accompanied by characteristic vitreous or retinal lesions. [Consensus score: 90% (strongly agree: 55%; agree: 35%; neutral: 10%; disagree: 0%; strongly disagree 0%)].*

### Section 2. Clinical diagnosis

**Fig. 1:**
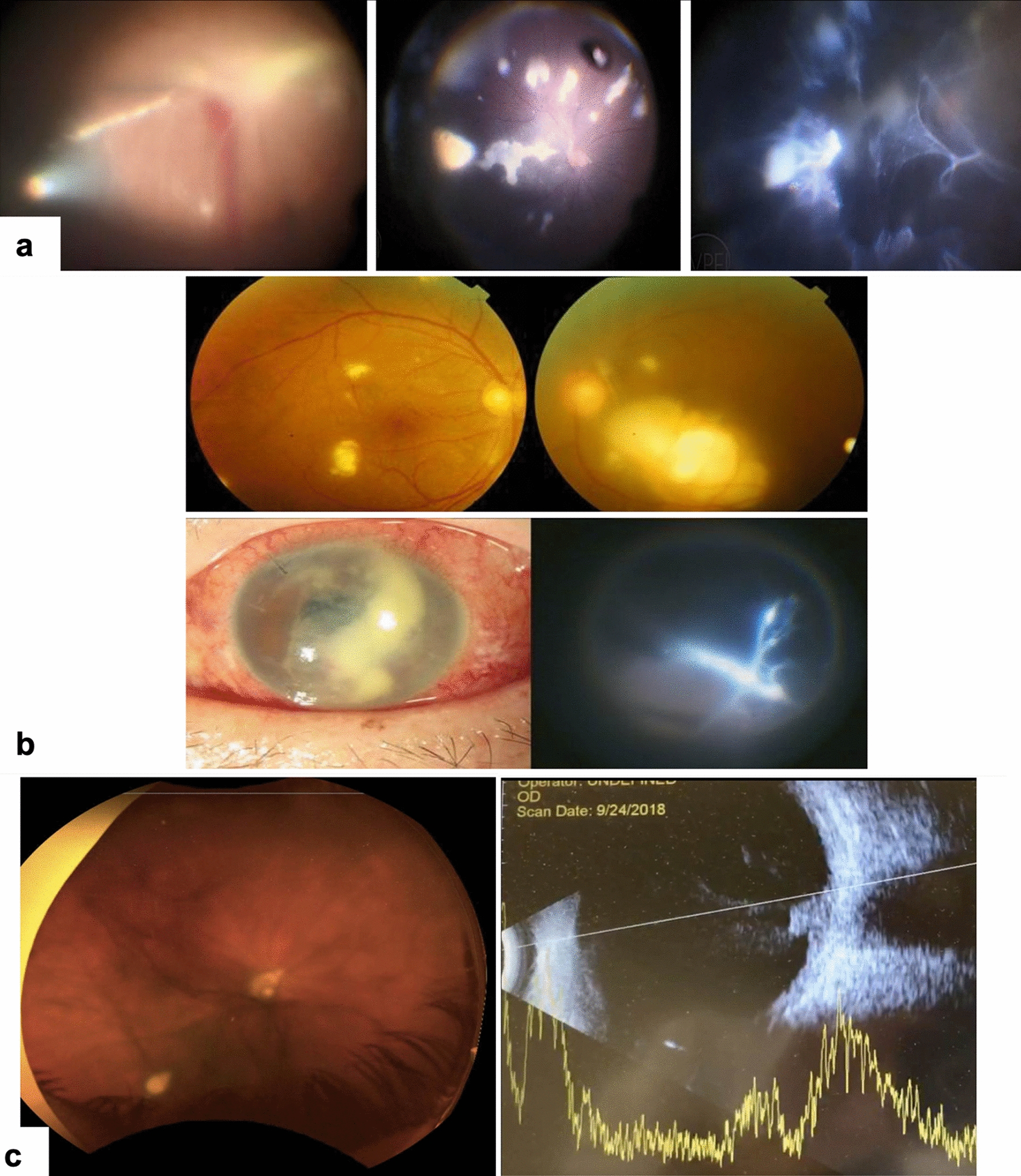
Typical signs of fungal endophthalmitis. **a** (Left) String of pearls; (Middle) Pre-retinal deposits; (Right) Vitreous membranes. **b** (Top panel) Bilateral endogenous fungal endophthalmitis. Multiple subretinal/ chorioretinal infiltrates and vitreous opacities, more in the left eye than in the right eye. Blood, urine, and vitreous fluid had *Candida albicans*. (Bottom panel) Unilateral endogenous fungal endophthalmitis. (Left) Slit-lamp examination shows corneal edema and anterior chamber exudation. Fundus examination shows tree-branch-like vitreous condensations. Blood and vitreous grew *Fusarium petroliphilum*. (Adapted with permission from [29].) **c** *Candida* endophthalmitis. (Left) Fundus-vitritis; two raised chorioretinal fungal lesions protruding into the vitreous. (Right) B-scan further delineating the lesions penetrating the Bruch’s membrane. (Adapted with permission from [57] CC-BY-NC)

The signs include variable degrees of lid edema, chronic conjunctival congestion, hypopyon, and intense vitritis. Typical signs of fungal endophthalmitis include nodular exudates over the iris and crystalline/intraocular lens (IOL) surface, vitreous exudates arranged like a string of pearls, and creamy white circumscribed chorioretinal lesions [29] (Fig. 1). Some prominent anterior and posterior segment signs of fungal endophthalmitis are listed hereunder (Table 2).

**Table 2:** Clinical features of fungal endophthalmitis

Anterior segment	Posterior segment
1. Conjunctival congestion 2. Dry appearing hypopyon 3. Yellowish-white nodular exudates on the iris/lens surface 4. Yellowish-white infiltrates at the corneoscleral wound of cataract surgery	1. Vitritis 2. Vitreous membranes 3. A string of pearl arrangement of vitreous exudates 4. Creamy white, circumscribed chorioretinal lesions

A history of using long-term topical corticosteroid and/or broad-spectrum antibiotic to reduce recurrent and persistent redness and inflammation is not uncommon. A relatively quiet-looking eye with mild vitreous haze in a patient on prolonged topical and systemic steroids after intraocular surgery should raise suspicion of fungal endophthalmitis. In post-cataract surgery endophthalmitis, the fungi may also be sequestered under the IOL/capsular bag, making it difficult to detect the fungi. Systemic investigations could be required; these include complete blood count, serum urea and electrolytes, liver function tests, peripheral blood culture, sputum and urine culture, chest radiogram, liver ultrasonogram, and transthoracic echocardiogram. The treating clinician decides the quantum of investigations based on the precipitating event and clinical suspicions.

In children, fungal endophthalmitis occurs more often after trauma. The infecting fungi are no different, and treatment is like treating endophthalmitis in adults, except that the children may require vitrectomy more often, and one should exercise caution in systemic antifungal therapy.

Ultrasonography (B-scan) is an important ancillary test to aid the clinical diagnosis. It quantifies vitreous exudation and structural changes of the retina and choroid [30]. It is useful in eyes with severe vitreous opacities, which limit the view of the retina. It also helps in making treatment decisions and prognosticating the treatment outcomes.

**Consensus Statement 2.1:** *Posterior segment signs are more apparent than the anterior segment signs in fungal endophthalmitis. [Consensus score: 75% (strongly agree: 40%; agree: 35%; neutral: 20%; disagree: 5%; strongly disagree 0%)].*

**Consensus Statement 2.2:** *Systemic investigations are essential in all fungal endophthalmitis. [Consensus score: 75% (strongly agree: 50%; agree: 25%; neutral: 20%; disagree: 5%; strongly disagree 0%)].*

### Section 3. Pathobiology

#### Pathology

Three key factors define the outcome of the infecting fungi: infectivity, pathogenicity, and virulence. The primary fungal pathogens can cause disease in immunocompromised patients. However, most pathogenic fungi are opportunistic and do not usually cause disease unless there are alterations in immune defenses. Infection occurs when fungi penetrate barriers (such as intact skin and mucous membrane linings) or breach the immunological defense (such as the host’s immunocompromised state or debilitating conditions). Fungi also gain access to the host tissues after penetrating trauma or inhalation. Exogenous endophthalmitis occurs when the mechanical barriers of the eye (eyelids, tear film, nonkeratinized squamous epithelium of conjunctiva and cornea) are breached by trauma, surgery, or the extension of fungal keratitis (including corneal perforation) [31, 32]. Endogenous infection spreads from the choroidal capillaries to the vitreous through disrupted Bruch’s membrane. The severity of the disease depends on the size of the inoculum, the magnitude of tissue destruction, the ability of the fungi to multiply in tissues, and the immunologic status of the host.

#### Microbiology

Common ocular pathogenic septate filamentous fungi are *Aspergillus*, *Fusarium*, and the common pathogenic yeast is *Candida* species. Aspergillosis occurs in people with chronic pulmonary diseases, organ transplants (liver, renal, and bone marrow), leukemia, and drug abuse. The fungus usually gains access to the eye as it spreads from the lungs to the choroid and invades the retinal and choroidal vessel walls. *Fusarium* endophthalmitis usually occurs as an extension of the nonhealing corneal ulcer (*Fusarium* keratitis is common) [31]. There was a higher incidence of *Mucor* infection in endogenous endophthalmitis during the SARS-CoV-2 pandemic in India [33]. Table 3 lists the common endophthalmitis-causing fungi and associated risk factors.

**Table 3:** Common ocular fungi and risk factors causing endophthalmitis. [21, 34, 35]

Category	Fungi	Risk factors
Post-procedure	*Aspergillus* spp. *Candida* spp. *Fusarium* spp.	• Cataract surgery • Intraocular injections • Contaminated surgical supply • Extension of fungal keratitis
Post-traumatic	*Fusarium* spp. *Aspergillus* spp. *Candida* spp.	• Penetrating ocular trauma • Vegetative matter injuries • Rural injuries
Endogenous	*Candida* spp*.* *Cryptococcus* spp. *Histoplasma* spp. *Aspergillus* spp*.*	• Hematogenous spread from systemic fungemia • Intravenous drug use • Immunosuppression

**Fig. 2:**
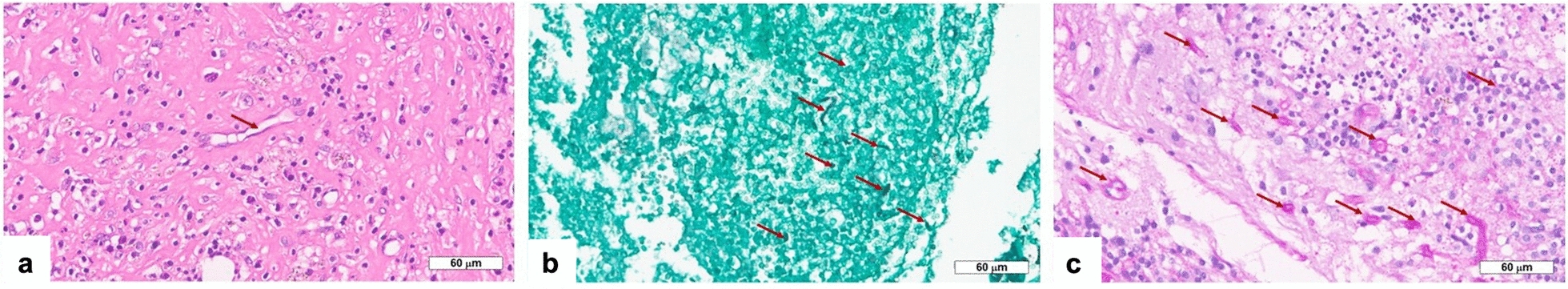
Photomicrograph depicting inflammatory cells along with septate fungal filaments in the intraocular contents (red arrows). (**a**) H&E, (**b**) GMS and (**c**) PAS staining (400×). H&E, hematoxylin and eosin; GMS, gomori methenamine silver; PAS, periodic acid Schiff

Histopathology of formalin-fixed tissue and microbiology of ocular fluid are the two primary sources of laboratory confirmation. In histopathology, the inflammatory cells are best seen by hematoxylin and eosin (H&E) stains. The fungal hyphae appear refractile to pale acidophilic filaments, thin or broad, septate or aseptate, and with or without branching. These hyphae are highlighted by special stains like periodic acid Schiff (PAS) and gomori methenamine silver (GMS) (Fig. 2). The characterization of fungi on histopathology is limited; hence, further ancillary techniques or microbiological culture correlation are needed [36].

**Fig. 3:**
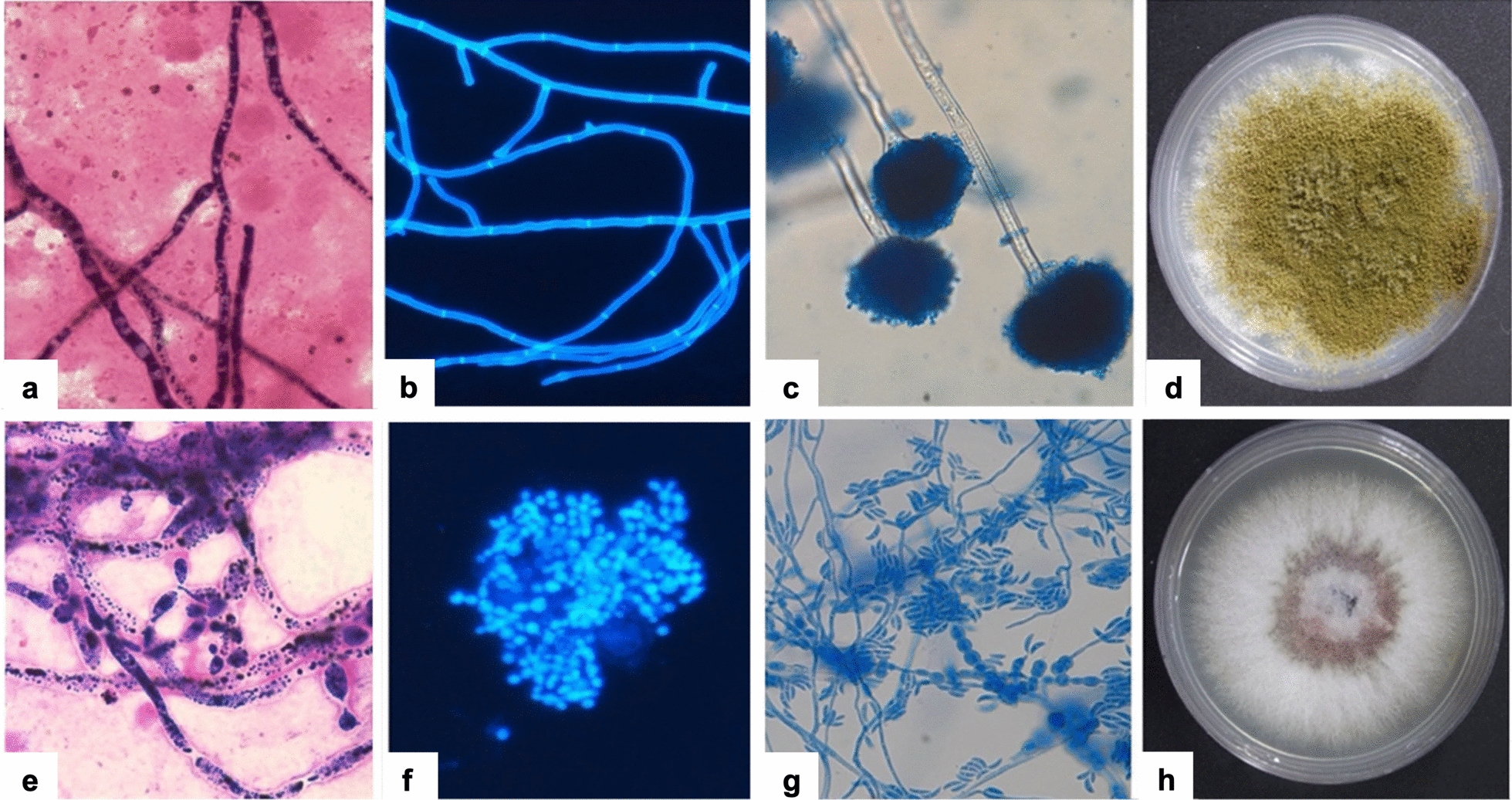
Microbiology of common fungi causing endophthalmitis. **a**, **b** Microscopy—septate branching filaments (Gram stain 1000×); Fluorescent, septate, branching fungal filaments (CFW, 400×) in vitreous samples were later identified as *Aspergillus* spp. (Adapted with permission from [87]). **c**, **d** *Aspergillus flavus*. **c** Microscopy of culture-LPCB mount showing septate, hyaline hyphae with rough-walled conidiophores terminating in globose vesicles bearing phialides (400×). **d** The culture on SDA shows characteristic granular, yellow-green colonies, typical of *A. flavus*, after 5 days of incubation at 25 °C. **e, f** *Candida albicans*. **e** Microscopy—Gram stain of culture showing Gram-positive, oval to budding yeast cells with pseudohyphae (1000×). **f** CFW Microscopy—stain highlighting budding yeast cells and occasional germ tubes under fluorescence microscopy (400×). **g, h** *Fusarium solani*. **g** Microscopy of culture-LPCB mount showing hyaline, septate hyphae with abundant sickle-shaped macroconidia and a blunt apical cell (400×). **h** Culture on SDA incubated at 25–30 °C showing fast-growing, cottony colonies with a white surface that later becomes pink to violet with a pale reverse. (this figure is contributed by Dr. Joveeta Joseph, original author). CFW, calcofluor white; LPCB, lactophenol cotton blue; SDA, sabouraud dextrose agar

Microbiological confirmation of fungi includes direct microscopy and culture. Direct microscopy using lactophenol cotton blue (LCB) and calcofluor white (CFW) is quicker. The color changes with the special stains help: Blue with LCB and fluorescent with CFW. Sabouraud dextrose agar (SDA) and potato dextrose agar (PDA) are selective media for fungi; SDA and PDA are incubated at 25 °C for 2–4 weeks. The chocolate agar/blood agar/brain heart infusion broth could also be used. Fungi are slow-growing organisms; cultures may take up to two weeks (Fig. 3).

Minimum fungicidal concentration (MFC) in fungal infection is like minimum inhibitory concentration (MIC). MIC is the lowest concentration of an antimicrobial agent that prevents ≥ 95% of visible growth of a microorganism, usually bacteria. MFC is the lowest drug concentration that achieves ≥ 98% killing of a particular fungus. While MIC remains the standard endpoint for susceptibility testing due to ease of use and clinical relevance in most settings, MFC has shown better correlation with clinical outcomes in severe fungal infections especially in immunocompromised hosts, deep-seated infections (e.g., meningitis, endophthalmitis), and where fungicidal action is preferred or critical [37].

Resistance to antifungal drugs occurs through several mechanisms. It includes (1) nonsynonymous point mutations within the gene encoding the target enzyme (leading to alteration in the amino acid sequence), (2) increased expression of the target enzyme through increased transcription of the gene encoding it, (3) decreased concentrations of the drug within the fungal cells due to drug efflux, and (4) changes in the biosynthetic pathway resulting in reduced production of the target of the antifungal agents [38–40]. Biofilm formation is one of the major causes of resistance. The potential to form biofilms has been demonstrated in some ocular fungi (such as *Aspergillus fumigatus*, *Candida albicans*, *Fusarium solani*, *Cladosporium sphaaerospermum*, and *Acremonium implicatum*) [41, 42].

In recent years, molecular microbiology techniques and technologies have significantly advanced for rapid detection of infection. These techniques are highly sensitive and specific enough to complement the conventional methods. The most widely used molecular techniques are the polymerase chain reaction (PCR) and real-time PCR. PCR detects the DNA of microorganisms in the intraocular fluids; the primers are either uniplex or multiplex for detecting a particular organism or a group of organisms. Pan-fungal PCR is useful in diagnosing fungal endophthalmitis [43]. Next-generation sequencing (NGS) is a high-throughput, culture-independent technique. It allows an unbiased approach to detecting pathogens. Targeted NGS provides better sensitivity and specificity. Studies in India have reported a large proportion of polymicrobial infections in culture-negative endophthalmitis [44, 45]. Investigators have shown similar results after targeted metagenomics and Nanopore targeted sequencing (NTS). NGS can identify virulence-associated genes that could help identify resistant pathogens to guide medical therapy [46–48].

**Consensus Statement 3.1:** *Fungal infection occurs more often in immunocompromised people. [Consensus score: 95% (strongly agree: 50%; agree: 45%; neutral: 5%; disagree: 0%; strongly disagree 0%)].*

**Consensus Statement 3.2:** *Common ocular pathogenic septate filamentous fungi are Aspergillus, Fusarium, and the common pathogenic yeast is Candida. (Consensus score: 100% (strongly agree: 75%; agree: 25%; neutral: 0%; disagree: 0%; strongly disagree 0%)].*

**Consensus Statement 3.3:** *The microbiology confirmation of fungi includes direct microscopy and culture. [Consensus score: 100% (strongly agree: 75%; agree: 25%; neutral: 0%; disagree: 0%; strongly disagree 0%)].*

**Consensus Statement 3.4:** *The MFC should be used in fungal infection. [Consensus score: 75% (strongly agree: 45%; agree: 30%; neutral: 20%; disagree: 0%; strongly disagree 5%)].*

**Consensus Statement 3.5:** *In all culture-negative endophthalmitis, molecular microbiology work-up, such as NGS, helps identify the infecting fungi. [Consensus score: 95% (strongly agree: 55%; agree: 40%; neutral: 5%; disagree: 0%; strongly disagree 0%)].*

### Section 4. Management

Vitrectomy is often required in infective fungal endophthalmitis for several reasons. It plays diagnostic, therapeutic, and complication-management roles. It physically reduces infectious load (debris and fungal elements), inflammatory cells, necrotic tissue, and retina-damaging toxins. It helps drug penetration, prevents/minimizes structural damage, clears the media opacities, and helps restore vision. In a large Indian series of 730 culture-proven cases of fungal endophthalmitis, nearly all patients underwent vitrectomy [21]. Vitrectomy is also indicated for treating complications of fungal endophthalmitis, such as persistent vitreous opacities, epiretinal membrane, and retinal detachment [49].

Patients with fungal endophthalmitis benefit from early vitrectomy. [50, 51]. Unlike the Endophthalmitis Vitrectomy Study recommendations for conservative vitrectomy in bacterial endophthalmitis (i.e., 50% vitreous removal) [3], fungal infections often require more extensive vitreous clearance [52]. Where it is safe, induction of a limited posterior hyaloid separation over the posterior pole but staying short of the periphery can be attempted. In pseudophakic eyes, clearance of the anterior vitreous is required, as exudates often accumulate near the ciliary sulcus, and inadequate clearance can lead to persistent or recurrent inflammation. Endoscopic vitrectomy or temporary keratoprosthesis can be considered when corneal opacity limits visualization [53]. Apparently, the anatomical and visual outcomes are better after endoscopic vitrectomy in the required situation.

Antifungal agents are the backbone of treating fungal endophthalmitis. Only a few antifungal molecules are used in ocular infections. Intravitreal antifungal agents are the mainstay of treatment, though it is equally important to administer them through other routes, such as topical and systemic, to treat all potential reservoirs of infection, maximize antifungal efficacy, and reduce the risk of recurrence. In fungal endophthalmitis, the vitreous is primarily involved, but the fungi may also reside in other ocular sites such as the anterior chamber, cornea, behind the ciliary body, and in the pseudophakic eye, around the IOL. Intravitreal antifungal agents deliver high drug concentration directly into the vitreous, topical agents treat infection in the anterior segment of the eye, and systemic antifungals potentiate the intravitreal drug concentration while treating the extraocular foci, if any. Multimodal treatment ensures a synergistic effect, improving the chances of eliminating the infection. A combination of intracameral and intravitreal voriconazole has been shown to be effective in managing fungal endophthalmitis; it should be considered, given the propensity of intra-capsular or peri-IOL sequestration [54].

Usually, more than one intravitreal injection is required. An Indian report of 730 culture-proven cases of fungal endophthalmitis reported that all patients needed vitrectomy and an average of two intravitreal antifungal agents [21]. The International Committee of Intraocular Inflammation Society recommends intravitreal antifungals only for mild vitritis and a combination of intravitreal antifungals and vitrectomy for moderate to severe vitritis [55].

Table 4 enumerates the essential differences in managing bacterial and fungal endophthalmitis. Systemic therapy, when begun after renal and liver function tests, must be continued for 4–6 weeks. A few studies have documented a faster reduction of inflammation after explantation of the IOL and posterior capsule in recalcitrant pseudophakic fungal endophthalmitis [56–59].

**Table 4:** Essential differences in the management of bacterial and fungal post-cataract endophthalmitis

Treatment modality	Post-cataract surgery bacterial endophthalmitis	Fungal endophthalmitis
Tap-Inj	Presenting vision ≥ HM	Not advised
Vit-Inj	Presenting vision ≤ LP Extent: 50% of vitreous	Used in most series of fungal endophthalmitis, irrespective of presenting vision
Intravitreal injections	Combinatorial antibiotics; One against GPC, one against GNB Repeat: at 36–72 h, if needed	One antifungal agent Repeat intravitreal injection is nearly a rule
Topical antimicrobials	Intensive	Intensive in post-procedural and post-traumatic cases
Systemic antimicrobials	No added value	Always in endogenous cases; optional in others
Corticosteroids	Intravitreal, topical, and systemic (based on institutional practice)	Intravitreal and systemic steroids are usually not used. Topical steroids could be used in some cases

*HM* = hand motions; *GNB* = gram-negative bacilli; *GPC* = gram-positive cocci; *IOL* = intraocular lens; *LP* = light perception; *PVA* = presenting visual acuity; *Tap-Inj* = vitreous tap (biopsy) + intravitreal injection; *Vit-Inj* = vitrectomy (includes biopsy) + intravitreal injection

#### Antifungal drugs

Five families of antifungals are extensively used to treat human fungal infections. The antifungal agents are dose-dependent, fungistatic, and fungicidal. The commonly used antifungal agents are: (1) polyenes (amphotericin B); (2) azoles, such as imidazoles (miconazole, econazole, ketoconazole), and triazoles (itraconazole, fluconazole, voriconazole, posaconazole); (3) echinocandins (caspofungin, micafungin, anidulafungin); (4) flucytosine, a pyrimidine analogue; and (5) allylamines (terbinafine). The three general mechanisms of action for antifungal agents are cell membrane disruption, cell division inhibition, and cell wall synthesis inhibition [60]. All these drugs inhibit the synthesis of or directly interact with ergosterol, the predominant component of the fungal cell membrane. The antifungal agents are usually fungistatic in the concentrations used in clinical practice but are fungicidal in higher concentrations. The primary route of administration of these agents is intravitreal injection, often supplemented with systemic (more often) and topical therapy [61] (Table 5).

**Table 5:** Antifungal agents and routes of administration in endophthalmitis

Antifungal agent			Intravitreal (0.1 mL)		Topical	Systemic (adult)	
Molecule	Class	Activity	Dose	Preparation		Dose	Toxicity
Amphotericin B	Polyene	Broad range	5 μg	50 mg + 10 mL DW Draw 0.1 mL + 0.9 mL NS Inject 0.1 mL	1.5 mg/mL (0.15%)	IV. 0.5–1.0 mg/kg	Kidney
Caspofungin	Echinocandins	*Aspergillus* *Candida*	50 μg	50 mg + 10 mL DW Draw 0.1 mL + 0.9 mL NS Inject 0.1 mL	5 mg/mL 0.5%	IV. 70 mg over 1 h^a^ IV. 50 mg over 1 h^b^	Liver
Ketoconazole	Imidazole	*Aspergillus* primarily	–	–	–	PO. 200 mg q 12 h	Liver
Fluconazole	Imidazole	*Candida* primarily	100–200 ug	2 mg/mL (0.05–0.1 mL)	2 mg/mL 0.2%	PO. 200–400 mg/day	Liver
Micafungin	Echinocandins	*Candia* *primarily*	25 μg	50 mg + 20 mL DW Draw 0.1 mL + 1.9 mL NS Inject 0.1 mL	–	IV. 100 mg q 24 h infusion	Liver
Posaconazole	Triazole	*Aspergillus* *Candida*	100 μg	300 mg + 30 mL DW Draw 0.1 mL + 0.9 mL NS Inject 0.1 mL	–	PO. 300 mg q 12 h^a^ PO. 300 mg q 24 h^b^	Liver
Natamycin	Polyene	Broad range	–	–	50 mg/mL*	-	-
Voriconazole	Triazole	Broad range	100 μg	200 mg + 20 mL DW Draw 0.1 mL + 0.9 mL NS Inject 0.1 mL	–	PO. 200 mg q 12 h IV. 6 mg/kg q 12 h^a^ IV. 4 mg/kg q 12 h^b^	Liver

*DW* = distilled water; *NS* = normal saline; *IV* = intravenous; *PO* = per os

^a^ represents loading dose; ^b^ represents maintenance dose; * represents commercially available

*Candida* species are generally susceptible to amphotericin B, fluconazole, and voriconazole. However, systemic amphotericin B achieves therapeutic intraocular concentrations in only a minority of cases, and it is typically reserved for azole-resistant infections. Fluconazole is a reasonable first-line option for susceptible *Candida* infections, given its lower cost, widespread availability, lower hepatic toxicity, and lack of need for therapeutic drug monitoring. Conversely, voriconazole has a broader spectrum, including activity against fluconazole-resistant species such as *Candida glabrata* and *Candida krusei*, and also covers filamentous fungi such as *Aspergillus* spp. [62–64].

Systemic therapy remains the mainstay of treatment in endogenous fungal endophthalmitis. If diagnosed early, when lesions are confined to the choroid (i.e., flat choroiditis) and have not breached the outer blood-retinal barrier, intravitreal therapy is typically unnecessary. However, adjunctive intravitreal antifungal therapy is warranted once lesions become raised (chorioretinitis) or involve the vitreous (endophthalmitis) [65].

Corticosteroids are not contraindicated in bacterial endophthalmitis. The Endophthalmitis Vitrectomy Study used oral prednisolone 30 mg twice daily, a day after the intravitreal antibiotics [3]. There are documented benefits of intravitreal dexamethasone in bacterial endophthalmitis [66–68]. But corticosteroids are not advised in fungal endophthalmitis for fear of worsening infection [30]. Intravitreal dexamethasone is generally not recommended in fungal endophthalmitis despite experimental and human studies of its possible benefits, as conflicting reports have also shown loss of eye when treated with intravitreal steroids [69–71].

While monotherapy (vitrectomy or intravitreal or systemic) might sometimes work, a combined therapy in fungal endophthalmitis is increasingly recognized as essential because of (1) poor ocular penetration of many systemic antifungals, (2) frequent biofilm formation on intraocular tissues of the IOL (pseudophakic eyes) that reduces the drug efficacy, (3) increasing drug resistance, and (4) the severe inflammatory load resulting in fungal biomass and toxic debris in the vitreous cavity. Some of the new antifungal agents to treat invasive and refractory fungal infections are olorofim and terbinafine. Olorofim is the orotomide class of antifungals for the treatment of invasive mold infections. It inhibits dihydroorotate dehydrogenase, a key enzyme in the biosynthesis of pyrimidines. Olorofim has activity against many molds and thermally dimorphic fungi, including species that are resistant to azoles and amphotericin B [72]. Terbinafine is an allylamine antifungal agent and acts by inhibiting squalene epoxidase (an enzyme in the fungal ergosterol biosynthesis pathway) [73]. These antifungals are specifically useful in invasive filamentous fungal infection. These antifungals are currently reserved for refractory fungal infection and are often combined with intravitreal antifungal agents. Some of the examples are intravitreal voriconazole with systemic voriconazole + terbinafine, or systemic voriconazole + terbinafine + olorofim in *Fusarium* and *Lomentospora* infection [74, 75].

#### Treatment outcomes

**Fig. 4:**
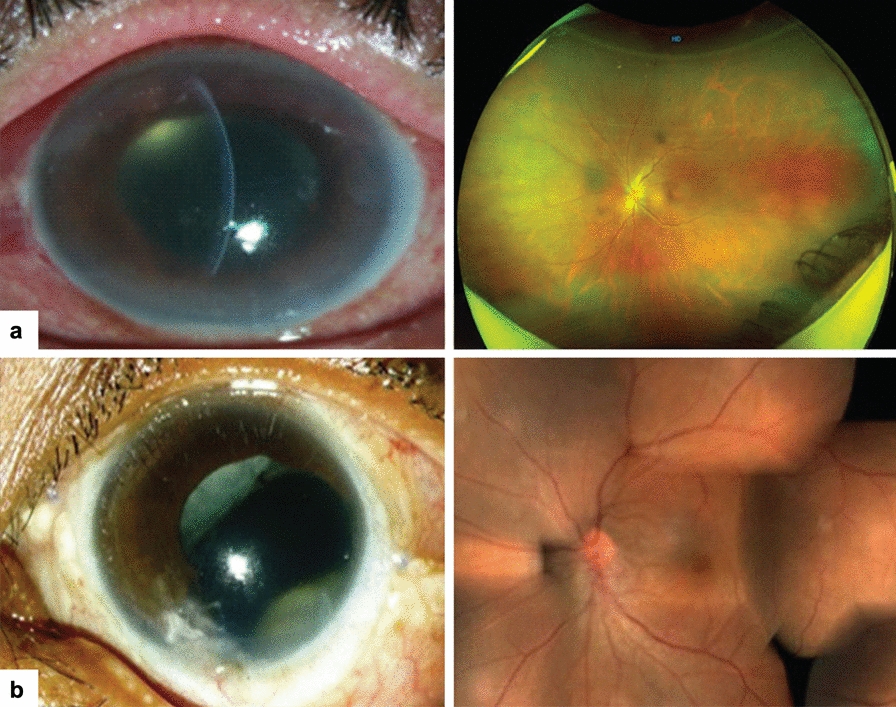
Treatment outcome in post-cataract and traumatic fungal endophthalmitis. **a** Post-cataract surgery endophthalmitis. At presentation, the eye was congested with corneal edema and a streak of hypopyon; the presenting vision was 20/100 (Left). After three vitreous surgeries, intravitreal antifungals, and intraocular lens explant, the final vision was 20/50. (Right) Vitreous grew *Aspergillus niger*. **b** Traumatic endophthalmitis. The patient was earlier treated with lensectomy-vitrectomy and was referred for recurrent infection. At presentation, the eye was aphakic with yellow-white exudates behind the iris. (Left) The presenting vision was finger count at two meters. After two additional vitrectomies and 14 intravitreal antifungals, the final vision was 20/60. (Right) Vitreous grew *Aspergillus flavus*. (Adapted with permission from [88] CC-BY-NC)

**Fig. 5:**
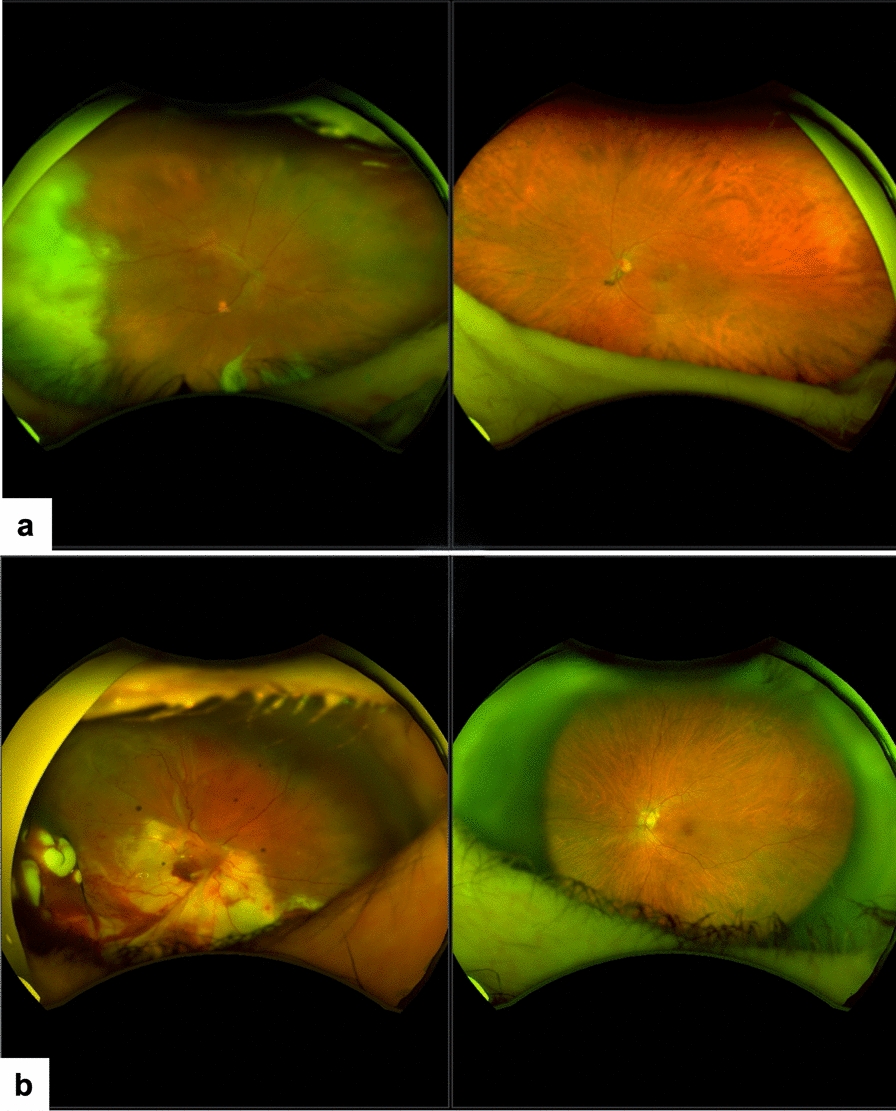
Treatment outcome in endogenous fungal endophthalmitis. **a** An 80-year-old patient presented with light perception vision in the right eye. Vitreous tap was negative for organisms and growth. Subsequent pars plana vitrectomy with sub-retinal exudate biopsy confirmed a *Candida albicans* infection, presumably secondary to antecedent colonoscopy. In addition to diagnostic and therapeutic vitrectomy, the patient was treated with intravitreal and systemic voriconazole, resulting in the resolution of subretinal exudate but minimal improvement in vision (hand movements at 50 cm). **b** A 72-year-old male on steroids for chronic obstructive pulmonary disease was referred from a tertiary referral hospital, where he had been admitted for the management of presumed endogenous endophthalmitis. The presenting vision was light perception. Previous pulmonary imaging revealed right upper lobe masses; bronchoscopy excluded malignancy and mycobacterial infection, though cultures were negative. Initial pars plana vitrectomy and vitreous microscopy, culture, and polymerase chain reaction were negative. A sub-retinal biopsy following pars plana vitrectomy (right and left eyes, 1 week following surgery in the right eye) confirmed Sc*edosporium apiospermum* infection. This was managed with intravitreal and systemic voriconazole. The patient subsequently demonstrated resolution of infection but developed a retinal detachment under oil with no improvement in vision

Generally, the visual outcome of fungal endophthalmitis is not as good as that of bacterial endophthalmitis [21]. A study from India that analyzed 730 cases reported that at least 10% of eyes needed therapeutic keratoplasty, and up to 7% were eviscerated. After treatment, the final (best corrected) visual acuity was > 20/400 in 30.5% of eyes, > 20/40 in 7.9% of eyes, and 12% (n = 88) of eyes lost light perception (Table 6). The outcomes could be better if the treatment is done sufficiently early and adequately. Some of the cases are illustrated in Figs. 4 and 5.

**Table 6:** Comparative treatment outcome of fungal and bacterial endophthalmitis in Indian studies. [21, 27, 76]

Vision after treatment	Postoperative		Post-trauma		Endogenous	
	Fungal [21] n = 342	Bacterial [68] n = 206	Fungal [21] n = 260	Bacterial [68] n = 182	Fungal [21] n = 128	Bacterial [27] n = 173
≥ 20/400 (%)	37.4	60.9	38.4	63.9	59.4	26.0
≥ 20/40 (%)	7.3	19.5	7.3	29.6	10.9	11.0
No light perception (%)	13.7	7.0	6.9	7.4	18.0	29.0

The recent identification of a novel fungal species, *Candida auris*, has developed into an international public health concern. *Candida auris* was first isolated in 2009 from the external ear canal of a 70-year-old patient from Japan [77]. Since then, infections due to *Candida auris* have been reported in over 40 countries, with mortality rates between 30 and 60%, largely secondary to significant and sometimes untreatable drug resistance to all known antifungal classes of drugs [78]. In 2022, Breazzano and colleagues described a case of endogenous panophthalmitis involving *Candida auris* in a 30-year-old immunocompromised male. The patient underwent enucleation and ultimately died despite treatment with intravitreal and systemic antifungals [79]. Diagnostic methods employed by laboratories frequently misidentify *Candida auris* as other organisms, making diagnosis, management, and infection control efforts challenging [80].

**Consensus Statement 4.1:** *Early and complete vitrectomy in fungal endophthalmitis results in a superior outcome. [Consensus score: 90% (strongly agree: 50%; agree: 40%; neutral: 10%; disagree: 0%; strongly disagree 0%)].*

**Consensus Statement 4.2: ***Treatment with antifungal agents is required *via* all routes—intravitreal, topical, and systemic. [Consensus score: 85% (strongly agree: 55%; agree: 30%; neutral: 10%; disagree: 5%; strongly disagree 0%)].*

**Consensus Statement 4.3:** *The IOL and posterior capsule explantation may be considered in recalcitrant pseudophakic fungal endophthalmitis. [Consensus score: 90% (strongly agree: 60%; agree: 30%; neutral: 10%; disagree: 0%; strongly disagree 0%)].*

**Consensus Statement 4.4:** *Intravitreal dexamethasone can be administered in fungal endophthalmitis. [Consensus score: 25% (strongly agree: 0%; agree: 25%; neutral: 40%; disagree: 20%; strongly disagree 15%)].*

**Consensus Statement 4.5:** *Systemic antifungal therapy should be initiated after baseline renal and liver function assessment and continued for 4–6 weeks. [Consensus score: 85% (strongly agree: 35%; agree: 50%; neutral: 15%; disagree: 0%; strongly disagree 0%)].*

### Section 5. Future developments

Indiscriminate use of antifungal agents and widespread agricultural antifungal exposure have resulted in resistance to one or more antifungal agents. The three principal concerns in antifungal therapy are: (1) physicians have less opportunity to switch therapy because susceptibility tests are not done routinely; (2) there is a limited choice of antifungal drugs; (3) there are no well‑defined endpoints.

Globally, the antifungal stewardship (AFS) has not received as much attention as antimicrobial stewardship. AFS is a coordinated effort within a healthcare setting to optimize the use of antifungal agents. It aims to improve patient outcomes, minimize adverse effects and reduce the risk of resistance. It calls for responsible use of antifungal molecules so that the most effective treatment can be administered while preserving the effectiveness of these medicines for future use. The Mycoses Study Group has made several recommendations for AFS, including engaging the management leadership group, increasing the accountability and responsibility of the health personnel, providing education and practical training, monitoring and surveillance, reporting, and feedback [81, 82].

Traditional culture takes longer, sometimes up to two weeks, for the fungi to grow. This eventually delays the diagnosis and treatment. Several newer avenues are now available commercially or are being experimented in different laboratories to expedite the detection of fungi. Some of these are inflammatory markers and newer molecular techniques. Some of these are described below.

#### Inflammatory markers

Galactomannan (GM) and 1,3 β‑D‑glucan (BDG) biomarkers have been successfully used to diagnose invasive fungal endophthalmitis using commercial ELISA Kits. 1,3‑BDG is a major polysaccharide cell wall component in many fungal species, including *Candida* and *Aspergillus* spp. An elevated BDG level in the vitreous fluid of patients with endogenous fungal endophthalmitis has been reported; it could be more sensitive [83].

GM is a cell wall component of *Aspergillus* spp. mainly. Its detection via enzyme immunoassay is part of the diagnostic criteria for invasive aspergillosis. A recent report has explored its use in diagnosing *A. fumigatus* in the vitreous sample and recommended its assay when the standard mycology is negative [84]. The data from India confirms significantly higher levels of vitreous GM in patients with culture‑proven fungal infections than in patients with non-infectious retinal disorders. These tests could be considered in conjunction with clinical and routine microbiological tests. The added advantage is the speed—the results of these tests are available within 2–3 h compared to several days of conventional culture [85].

#### Molecular methods

The molecular microbiology methods, in addition to PCR, real‑time PCR, and NGS, are matrix‑assisted laser desorption/ionization-time of flight (MALDI‑TOF), and peptide nucleic acid fluorescence in situ hybridization [44–48]. Recently, a rapid molecular diagnostic tool, RID-Myc assay, using CRISPR-Cas 12a technology, has been described; it is not yet commercially available [86].

Negative culture is a common limitation of the conventional microbiology workup of fungal endophthalmitis. Due to delayed presentation, the classic clinical characteristics could be masked. This could be resolved using molecular techniques, including PCR, Sanger sequencing, and targeted NGS [87]. NGS allows for unbiased, high-throughput detection of microbial DNA directly from intraocular specimens such as vitreous or aqueous humor. This culture-independent method can identify a broad range of pathogens including rare or fastidious fungi by analyzing genetic material even when organisms are nonviable or present in low abundance. As technology becomes more accessible and rapid, NGS has the potential to complement or even surpass standard diagnostics in complex or refractory cases of infectious endophthalmitis.

**Consensus Statement 5.1:** *Antifungal stewardship should be considered. [Consensus score: 95% (strongly agree: 60%; agree: 35%; neutral: 5%; disagree: 0%; strongly disagree 0%)].*

**Consensus Statement 5.2:** *Rapid diagnosis is required to institute early treatment. Fungal biomarkers like 1,3 β-D-glucan and galactomannan are valuable adjuncts for the early diagnosis of fungal endophthalmitis. [Consensus score: 90% (strongly agree: 25%; agree: 65%; neutral: 10%; disagree: 0%; strongly disagree 0%)].*

**Consensus Statement 5.3:** *Advanced molecular diagnostics such as PCR, NGS, and MALDI-TOF should be considered in suspected fungal endophthalmitis, especially when conventional microbiological methods are inconclusive. [Consensus score: 100% (strongly agree: 65%; agree: 35%; neutral: 0%; disagree: 0%; strongly disagree 0%)].*

## Results of voting and discussion

Table 7 provides a summary of the key consensus statements along with the corresponding voting results.

**Table 7:** Results of the voting on the consensus statements of fungal endophthalmitis

Section	Consensus statement	C score (%)	Strongly agree (%)	Agree (%)	Neutral (%)	Disagree (%)	Strongly disagree (%)
1. Disease entity							
1.1	Fungal infections of the eye occur less frequently than bacterial infections. It is usually a delayed presentation	**100**	80	20	0	0	0
1.2	Endogenous fungal endophthalmitis is as common as exogenous endophthalmitis. The common systemic diseases are the liver, the lungs (pneumonia), and the endocardium	*50*	15	35	15	35	0
1.3	Acute fungal endophthalmitis after an intraocular procedure is also a possibility	**95**	40	55	5	0	0
1.4	Traumatic fungal endophthalmitis usually results from vegetative matter or soil injuries and presents earlier than other forms of fungal endophthalmitis	**100**	70	30	0	0	0
1.5	Vitritis is the most consistent clinical presentation in endogenous fungal endophthalmitis and may be accompanied by characteristic vitreous or retinal lesions	**90**	55	35	10	0	0
2. Clinical diagnosis							
2.1	Posterior segment signs are more apparent than the anterior segment signs in fungal endophthalmitis	**75**	40	35	20	5.0	0
2.2	Systemic investigations are essential in all fungal endophthalmitis	**75**	50	25	20	5	0
3. Pathobiology							
3.1	Fungal infection occurs more often in immunocompromised people	**95**	50	45	5	0	0
3.2	Common ocular pathogenic septate filamentous fungi are *Aspergillus*, *Fusarium*, and the common pathogenic yeast is *Candida*	**100**	75	25	0	0	0
3.3	The microbiology confirmation of fungi includes direct microscopy and culture	**100**	75	25	0	0	0
3.4	The minimum fungicidal concentration should be used in fungal infection	**75**	45	30	20	0	5
3.5	In all culture-negative endophthalmitis, molecular microbiology work-up, such as NGS, helps identify the infecting fungi	**95**	55	40	5	0	0
4. Management							
4.1	Early and complete vitrectomy in fungal endophthalmitis results in a superior outcome	**90**	50	40	10	0	0
4.2	Treatment with antifungal agents is required via all routes—intravitreal, topical, and systemic	**85**	55	30	10	5	0
4.3	The intraocular lens and posterior capsule explantation may be considered in recalcitrant pseudophakic fungal endophthalmitis	**90**	60	30	10	0	0
4.4	Intravitreal dexamethasone can be administered in fungal endophthalmitis	*25*	0	25	40	20	15
4.5	Systemic antifungal therapy should be initiated after baseline renal and liver function assessment and continued for 4–6 weeks	**85**	35	50	15	0	0
5. Future developments							
5.1	Antifungal stewardship should be considered	**95**	60	35	5	0	0
5.2	Rapid diagnosis is required to institute early treatment. Fungal biomarkers like 1,3 β-D-glucan and galactomannan are valuable adjuncts for the early diagnosis of fungal endophthalmitis	**90**	25	65	10	0	0
5.3	Advanced molecular diagnostics such as PCR, NGS, and MALDI-TOF should be considered in suspected fungal endophthalmitis, especially when conventional microbiological methods are inconclusive	**100**	65	35	0	0	0

Consensus score (C score) was defined as the value of the summation of the ‘strongly agree’, and ‘agree’ percentages; C score ≥ 75% was considered ‘consensus achieved’, and C score < 75% was ‘consensus not reached’. Only two statements were ‘consensus not achieved’ (unbold and underlined). Bold indicates statements which were ‘consensus achieved’

*PCR* = polymerase chain reaction; *NGS* = next-generation sequencing; *MALDI-TOF* = matrix‑assisted laser desorption/ionization-time of flight

Fungal infections of the eye are less frequent than bacterial infections, but they are more serious. At the same time, research on fungal infections is generally less extensive than research on bacterial infections. There are several reasons, including (1) it was always considered a niche problem (occurring in immunocompromised and transplant recipients) and never perceived as a public health problem (for example, tuberculosis or plague), (2) more challenging to diagnose that could result in under-reporting and hence does not attract the funding agencies as they do not view a priority disease due to low incidence reports, (3) the fungi being eukaryotic organisms like human cells makes it harder to develop antifungal drugs that are effective against fungi but not toxic to humans (unlike bacteria, prokaryotic microorganisms, are biologically more distinct from humans, making selective targeting easier), and (4) low levels of public and institutional awareness. Hence, there are fewer large studies and no randomized clinical trials for fungal endophthalmitis. This communication collated the available literature and surveyed twenty-four world experts to create a consensus and understand the controversies in managing fungal endophthalmitis. In 18 of 20 (90%) statements, there was good agreement (> 75% ‘agree’ to ‘strongly agree’); this included epidemiology, the clinical signs, pathobiology, management, and future developments. In two statements, there were fewer agreements (< 75%); these included (1) the relative incidence of exogenous and endogenous endophthalmitis and (2) the utility of intravitreal dexamethasone. (see Table 7) The following could be explanations for these varied opinions. (1) Given that exogenous (after intraocular procedures and trauma) fungal endophthalmitis is more often reported from Asia, and endogenous fungal endophthalmitis is more often reported from Europe, North America, and Oceania, the opinion on the frequency and the signs is bound to vary. (2) The corticosteroids suppress the immune response, which can worsen the fungal infection by reducing the host immune response. Intravitreal corticosteroid (Consensus Statement 4.4) also attracted three ‘strongly disagree’ answers. While it still does not have universal acceptance, in selected and carefully monitored situations, corticosteroids might be considered adjunctively after antifungal therapy is established and fungal infection is possibly controlled. The other ‘strongly disagree’ answer was to the routine MFC test (Consensus Statement 3.4). However, the MFC test should be a standard microbiology work-up, considering growing resistance to commonly used antifungal agents.

There are 1.5 to 5 million species of fungi that can grow almost anywhere e.g., in water, soil, plants, and animals. The primary pathogenic fungi usually have an environmental reservoir [88]. Opportunistic pathogens take advantage of debilitated or immunocompromised hosts to cause infection. Unlike fungal keratitis [89], the global burden of fungal endophthalmitis is not reported. Assessment of the worldwide burden and epidemiologic trends is critical to prioritizing prevention strategies, diagnostic modalities, and therapeutic interventions. However, quantifying the global burden of fungal endophthalmitis has several challenges, as has already been stated. Given the low incidence and regional variations, there is no well-defined protocol for diagnosing and treating fungal endophthalmitis. A randomized clinical trial is not feasible for fungal endophthalmitis. Analyzing real-world data and sourcing real-world evidence is one modality to create an appropriate management strategy [90, 91].

**Fig. 6:**
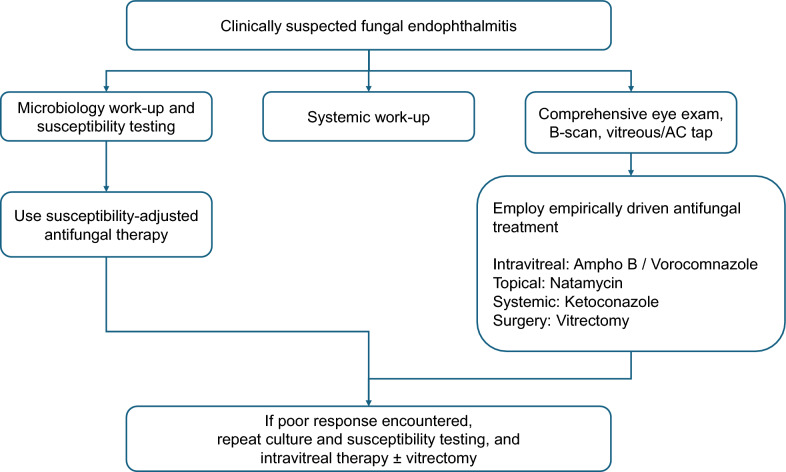
Suggested management algorithm of fungal endophthalmitis

The current consensus, derived from expert clinical experience and a review of available literature, aimed to address the main hurdles ophthalmologists face in diagnosing and managing this vision-threatening condition. This guideline emphasizes the importance of clinical suspicion following intraocular surgery, trauma, and in immunosuppressed people. The panel recommends combining direct microscopy, culture, and molecular techniques in culture-negative cases. The limited availability of effective antifungal agents with good intraocular penetration and safety profiles further complicates the treatment of fungal endophthalmitis. The panel supports the early use of intravitreal antifungals. The higher frequency of *Candida* species in endogenous endophthalmitis and *Aspergillus* species in exogenous endophthalmitis calls for different antifungal strategies. See Fig. 6 for a suggested treatment strategy for fungal endophthalmitis.

## Conclusion

Fungal endophthalmitis remains a formidable diagnostic and therapeutic challenge in ophthalmology. It is primarily due to its insidious onset, limited diagnostic tools, and the complex nature of fungal pathogens. There is a clear consensus that exogenous infection occurs in tropical and subtropical climates, that endogenous infection occurs after systemic infection in immunocompromised people and drug users, and that early recognition, microbiological confirmation, and early appropriate antifungal therapy result in better diagnosis. A significant area of controversy is corticosteroid therapy. Corticosteroids in fungal infections are not considered for fear of the risk of immune suppression and exacerbation of fungal proliferation, yet some clinicians argue for their cautious use in select cases to mitigate damaging inflammatory responses once the fungal infection is controlled. Newer diagnostic techniques [86] and newer antifungal agents [72–75] hold promise. While a randomized clinical trial is the ideal solution, a more nuanced understanding of the disease spectrum, multidisciplinary research, and real-world evidence are critical.

## Data Availability

All data generated or analyzed during this study are included in this published article and its supplementary information files.

## Abbreviations

AAPPO: Asia-Pacific Academy of Professors in Ophthalmology
AFS: Antifungal stewardship
APSOII: Asia-Pacific Society of Ocular Inflammation and Infection
APVRS: Asia-Pacific Vitreo-retina Society
BDG: β‑D‑glucan
CFW: Calcofluor white
GM: Galactomannan
GMS: Gomori methenamine silver
H&E: Hematoxylin and eosin
IOFB: Intraocular foreign body
IOL: Intraocular lens
LCB: Lactophenol cotton blue
MALDI-TOF: Matrix‑assisted laser desorption/ionization-time of flight
MFC: Minimum fungicidal concentration
MIC: Minimum inhibitory concentration
NGS: Next-generation sequencing
PAS: Periodic acid Schiff
PCR: Polymerase chain reaction
PDA: Potato dextrose agar
SDA: Sabouraud dextrose agar
